# Neurolymphomatosis of the median nerve, optic nerve, L4 spinal nerve root and cauda equina in patients with B-cell malignancies: a case series

**DOI:** 10.1186/s13256-021-02714-8

**Published:** 2021-03-26

**Authors:** S. Alazawi, H. Elomri, R. Taha, M. Bakr, M. T. Abdelhamid, L. Szabados, M. Yassin, H. El Sabah, K. Aboudi, A. Ellahie, A. Fadul, A. Gameil, A. Al Battah, L. J. Fernyhough

**Affiliations:** 1 Department of Hematology/Oncology, National Center for Cancer Care and Research, Hamad Medical Corporation, Doha, Qatar; 2 Department of Radiology, National Center for Cancer Care and Research, Hamad Medical Corporation, Doha, Qatar; 3 Medicine, Mansoura University, Mansoura, Egypt; 4 Weill Cornell Medicine, Doha, Qatar

**Keywords:** Burkitt lymphoma, Renal transplant, Neurolymphomatosis, Median nerve palsy, Post-transplant lymphoproliferative disorder (PTLD), B cell-acute lymphoblastic leukemia (b-ALL), Optic nerve lesion, Diffuse large B-cell lymphoma, Cauda equina syndrome, L4 spinal nerve root

## Abstract

**Background:**

Neurolymphomatosis is rare. Neoplastic lymphocytes are seen to invade nerves (cranial or peripheral), nerve roots or other related structures in patients with hematological malignancy. It is a separate entity from central nervous system lymphoma. Neurolymphomatosis has most commonly been described in association with B-cell non-Hodgkin lymphoma. Neurolymphomatosis in the context of Burkitt lymphoma and the post-renal transplant setting has not been described before.

**Case reports:**

We report for the first time in the Arabian Gulf countries and nearby Arab states four cases of neurolymphomatosis (one Asian, and the other 3 are from Arabic nationals) occurring between 2012 and 2017 involving the median nerve, optic nerve, nerve root and cauda equina in patients with Burkitt lymphoma, Philadelphia chromosome-positive B-cell acute lymphoblastic leukemia and diffuse large B-cell lymphoma.

**Conclusions:**

Neurolymphomatosis is rare and can be difficult to diagnose by biopsy but reliably confirmed by a combined imaging approach. Prior treatment with high-dose dexamethasone might suppress 18F-fluorodeoxyglucose (FDG) activity and decrease the sensitivity of positron emission tomography/computed tomography (PET/CT). The prognosis is generally poor but using high-dose methotrexate as well as high-dose chemotherapy and autologous stem cell transplantation may be an effective way to treat neurolymphomatosis.

## Background

Neurolymphomatosis (NL) is the term for nerve infiltration by neurotropic neoplastic cells in non-Hodgkin lymphoma (NHL) or acute leukemia [1]. It is rare, estimated to occur in only 0.2% of NHLs. Most NL cases are caused by B-cell disease, predominantly diffuse large B-cell lymphoma [2–9], although it has infrequently been described in leukemias and T-cell lymphomas [10–13]. The presenting symptoms and signs are diverse, including radicular pain, cranial nerve palsy, weakness, sensory change and, less commonly, Guillain-Barré and cauda equina syndromes [1, 2, 13, 14]. There may be long delays in diagnosis due to unusual symptomatology and difficulties in obtaining biopsy material from nerves [1, 2]. Biopsy of involved neurological structures remains the gold standard for diagnosis but has limitations due to difficulty accessing the target nerves as well as the potential for permanent neurological damage. The diagnostic sensitivity of magnetic resonance imaging (MRI) is reported to be 40–80% [1, 2], and the finding of nerve or nerve root enlargement or thickening, with or without contrast enhancement, can be seen on MRI scans [15–18]. PET-CT scanning can help by showing increased FDG uptake in the abnormal areas and by identifying an optimal site for biopsy, with a sensitivity of 87–100% for peripheral nerve lesions [13, 15, 19–26]. Cerebrospinal fluid (CSF) cytopathology evaluation may also be a useful tool in the 20–40% of NL patients who have concurrent meningeal dissemination [1, 2]. The use of flow cytometry and lymphocyte gene rearrangement studies for clonality assessment increases the diagnostic sensitivity and specificity [2]. Treatment of NL is often difficult, and there is no known standard treatment. Corticosteroids alone only provide short-term symptomatic relief [2]. Systemic chemotherapy with high-dose methotrexate with or without local radiotherapy has a variable response rate although many responses are not durable. Although the addition of rituximab (R) to CHOP (cyclophosphamide, doxorubicin, vincristine and prednisolone) chemotherapy has been shown to significantly improve the survival in other patients with diffuse large B-cell lymphoma (DLBCL) [27, 28], other reported cases suggest that rituximab does not improve the outcome in NL [29]. Although early detection of the disease, optimal systemic chemotherapy, corticosteroids and local radiotherapy may significantly reduce morbidity and improve outcomes, relapse is the rule in most reported cases, suggesting that better treatment strategies are required for NL.

Here we describe four cases with NL with disparate presenting features that together demonstrate different approaches to the diagnosis and treatment of NL as well as different outcomes.

## Case presentation

### Case 1: Burkitt lymphoma and neurolymphomatosis in the post-renal transplant setting

**Fig. 1:**
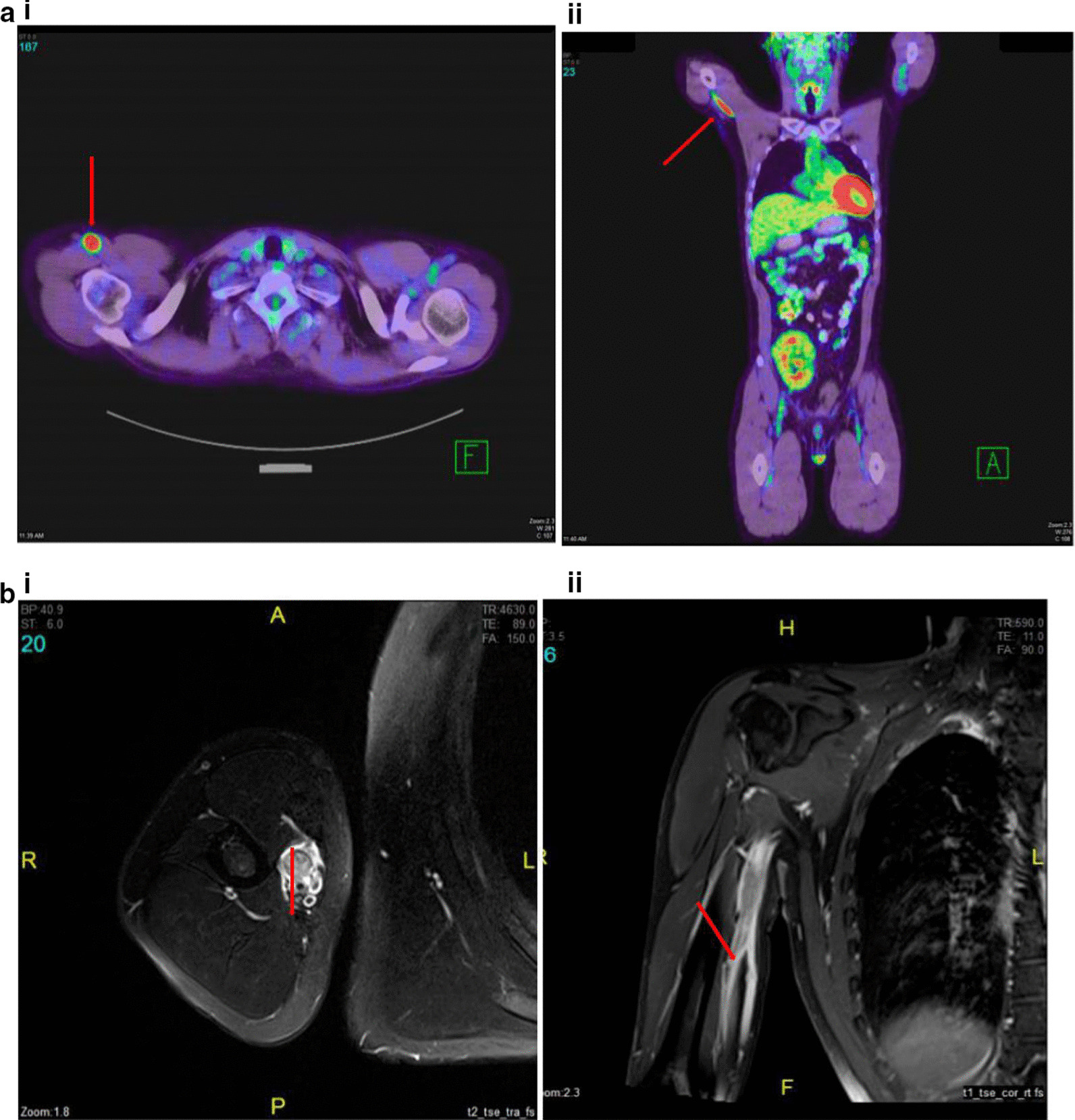
Case 1, radiological findings. **a** FDG PET-CT. Axial (i) and coronal (ii) planes show abnormally avid activity in the anatomical location of the right upper arm median nerve (arrows). **b** MRI axial T1-weighted post-contrast fat-saturated image (i). Coronal T1-weighted post-contrast fat-saturated image (ii). The right upper arm median nerve (arrows) is enlarged and displays abnormal signal intensity (low and high T1- and T2-weighted images, respectively) and abnormal post-contrast heterogeneous enhancement (**b**, i–ii)

**Fig. 2:**
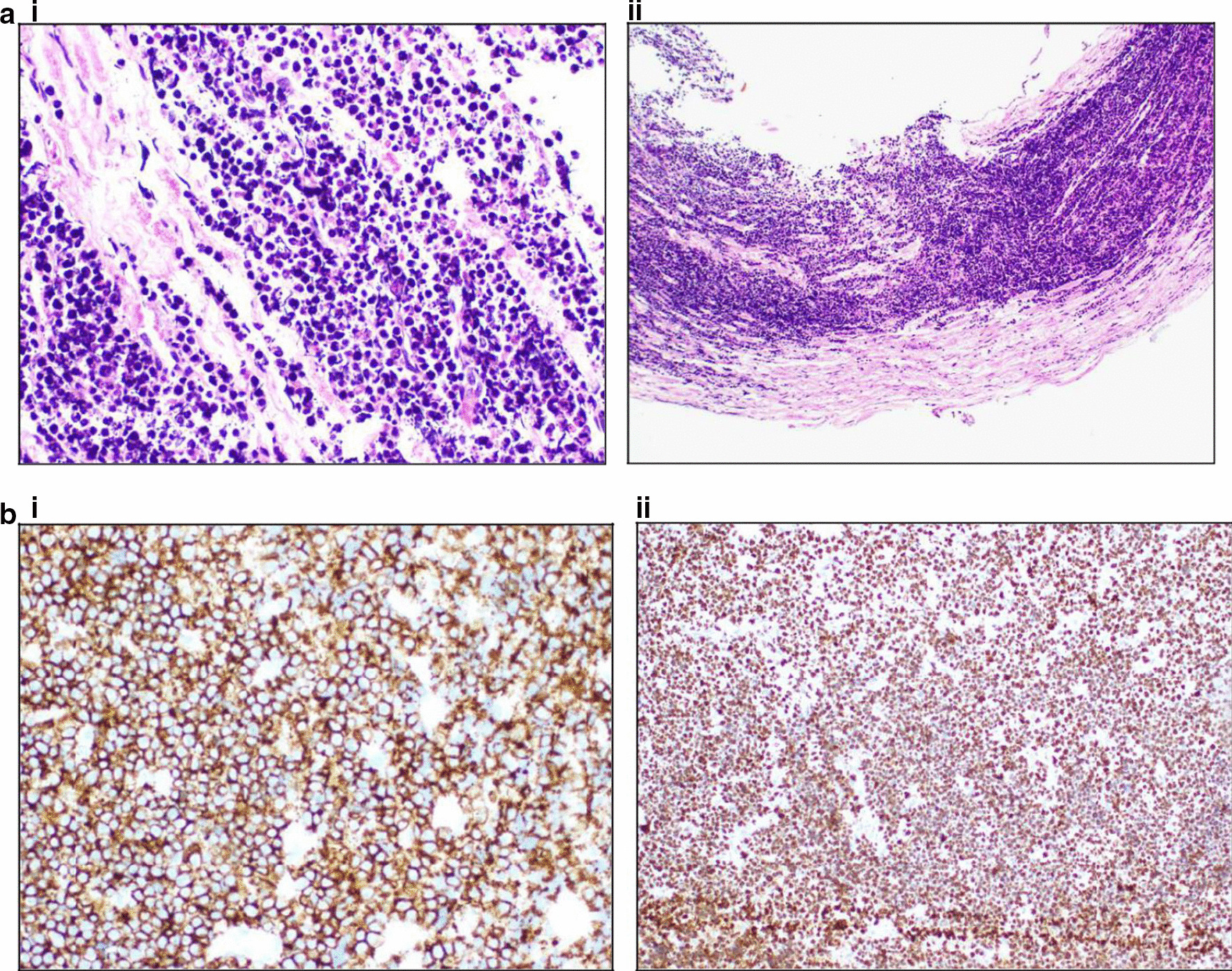
**a** (i) and (ii) Right median nerve biopsy. Neural tissue shows involvement by lymphoma cells compatible with Burkitt lymphoma. Positivity for CD20 **b**(i) and Ki67 (ii) is shown. Ki67 expression was 100%.

A 33-year-old Filipino man was diagnosed at our center with Burkitt lymphoma in July 2011. Disease staging revealed an Ann Arbor stage IA disease. The patient had undergone kidney transplantation in 2007 for renal failure secondary to chronic glomerulonephritis and had been on immune suppressive treatment with sirolimus 2 mg daily since that time. The patient was treated with a methotrexate-free regimen consisting of six cycles of R-CHOP and achieved a complete remission (CR). Three months post-CR, the patient presented with pain in the right axilla, extending to the thumb, index, middle fingers and median half of the ring finger. Hyperesthesia was present in the palm of the right hand, along the sensory distribution of the median nerve. The pain rapidly worsened and hampered daily activities involving the right upper limb. Clinical examination revealed the presence of an elongated hard mass, located on the proximal medial side of the right upper arm. There was also right thenar eminence atrophy, defective apposition of the right thumb and incomplete flexion of the index and middle fingers. The patient underwent a full-body PET-CT scan. This revealed abnormal FDG-avid activity in the anatomical location of the median nerve in the right upper arm (Fig. 1a i, ii). No other lesions were identified. MRI revealed enlargement of the median nerve with abnormal signal intensity after IV contrast administration (Fig. 1b i, ii). Cerebral spinal fluid (CSF) examination showed normal protein and glucose levels, mature lymphocytes 2–3 cells/mm [3] and absence of malignant cells. A bone marrow assessment showed no lymphomatous infiltration. Cytomegalovirus, Epstein-Barr virus and adenovirus polymerase chain reaction (PCR) tests were negative. Serology for hepatitis B and C virus was also negative. Electrophysiological studies showed reduced conduction velocities of the motor and sensory median nerve. A biopsy obtained from the right upper arm mass showed fragments of fibrous and neural tissue infiltrated by an abnormal population of cells resembling Burkitt lymphoma. Immunohistochemistry (IHC) performed on these cells revealed positivity for CD20, CD10 and Ki67 100% (Fig. 2) and negativity for CD3, CD5, Bcl2, Bcl6, TdT and cyclin D1 (not shown). The salvage therapy plan consisted of high-dose cytarabine and rituximab, 3 weekly for four cycles. After two cycles of salvage therapy, the patient showed a very good clinical response. An interim PET-CT indicated a 50% size reduction of the upper arm mass along with a reduced FDG uptake compared with the initial study.

While symptoms regressed significantly, the mass did not reduce further in size after the fourth cycle. Subsequent local radiotherapy (20 Gy) did not provide additional benefit. Following treatment, the patient decided to temporarily move to his home country and was lost to follow-up for 3 months. On his return, he presented with generalized lymphadenopathy and deteriorating general health. The patient and his family were informed of the rapid disease progression and limited therapeutic options. Two months after beginning palliative measures, the patient died.

### Case 2: B-cell acute lymphoblastic leukemia and neurolymphomatosis

**Fig. 3:**
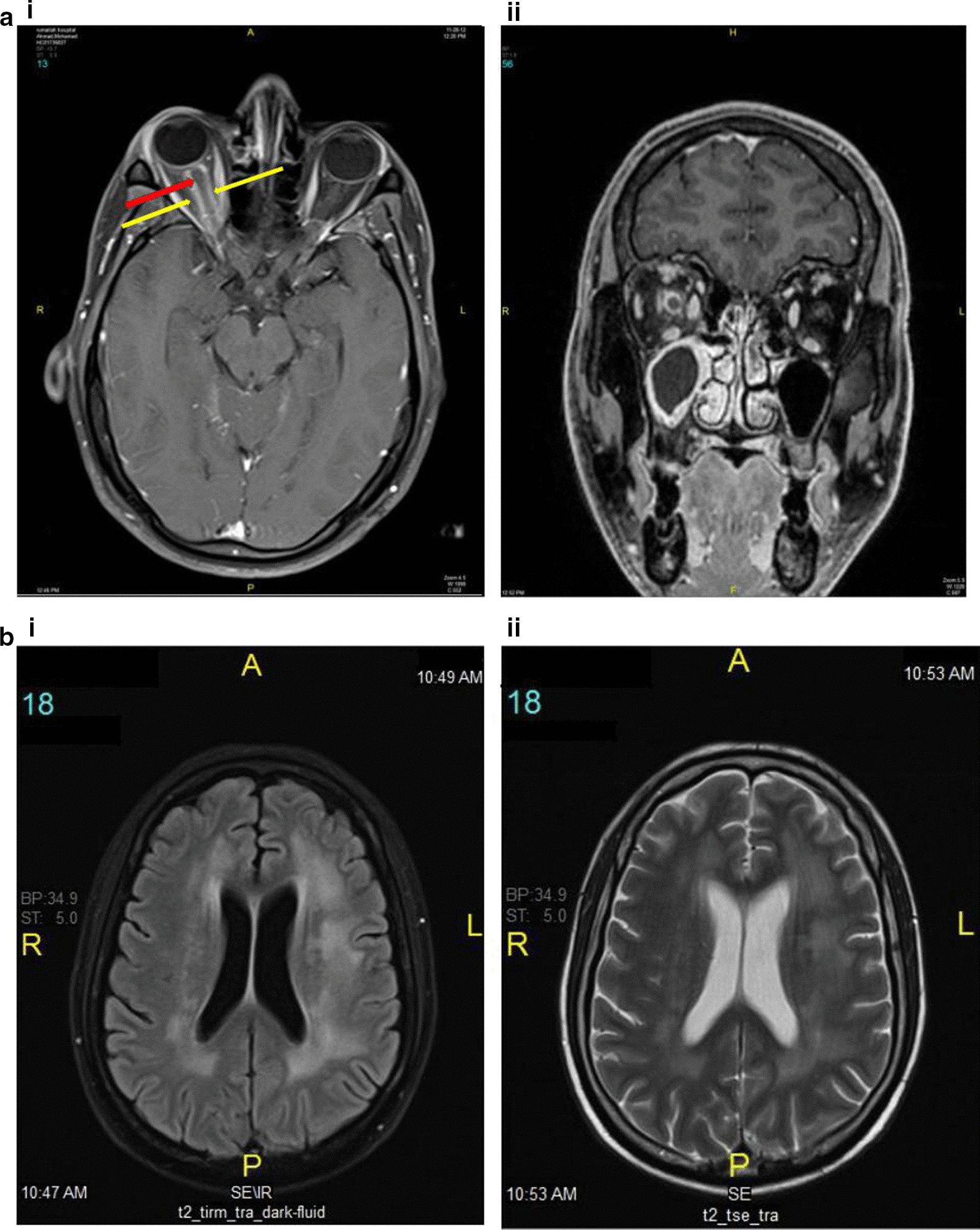
Case 2, radiological findings. **a** MRI brain at presentation. The right optic nerve is thicker than the left one (red arrow). Abnormal enhancement of the optic nerve sheath (tram-track sign) (yellow arrows) extends to the intraorbital and intracanalicular segments of the nerve. There is abnormal enhancement and stranding of the perioptic fat. Axial (i) and coronal (ii) T1-weighted post-contrast fat-saturated images are shown. **b** MRI brain at clinical progression. Newly developed multiple lesions in the periventricular white matter are noted. Two representative axial T1-weighted post-contrast fat-saturated images are shown

A 40-year-old Egyptian man presented in October 2010 with generalized lymphadenopathy, hepatosplenomegaly and marked leukocytosis (WBC 455,000/µL). Bone marrow showed a preponderance of BCR-ABL-positive lymphoblasts (60% of the total cellularity), consistent with pre-B acute lymphoblastic leukemia. The patient refused to receive a standard induction chemotherapy regimen and was initially treated with prednisolone and imatinib. A subsequent bone marrow reassessment showed no cytogenetic response. Three months post-diagnosis, the patient agreed to receive additional chemotherapy with Hyper-CVAD (hyper-fractionated cyclophosphamide, vincristine, doxorubicin and dexamethasone, alternating with high-dose methotrexate and cytarabine) together with dasatinib, a second-generation tyrosine kinase inhibitor. The patient achieved complete cytogenetic response after 3 months, but then refused any further chemotherapy except for oral dasatinib. Molecular remission was achieved after 6 months. CSF cytology was consistently negative, and no clinical finding suggesting central nervous system (CNS) involvement was present. In November 2012, the patient developed right eye swelling and blurred vision. MRI revealed a prominent right optic nerve with abnormal enhancement of the optic sheath involving the intra-orbital and intra-canalicular segments of the optic nerve (Fig. 3a). Cytology and flow cytometry analysis confirmed the presence of blasts in the CSF. Bone marrow disease was undetectable. Based on these findings, a diagnosis of isolated optic nerve neurolymphomatosis was made. The patient restarted Hyper-CVAD and bi-weekly intrathecal methotrexate. Right orbit radiation (20 Gy) therapy was given following chemotherapy. However, the patient’s vision rapidly deteriorated, and ultimately he became blind in his right eye despite slight radiological improvement of the right optic nerve thickening and enhancement. He remained stable for 20 months, but then developed slurred speech and right sensorineural hearing loss. At this time, MRI of his brain demonstrated multiple new lesions in the periventricular white matter (Fig. 3b). Salvage therapy with systemic high-dose methotrexate and ifosfamide along with intrathecal methotrexate was initiated. Severe hematological and non-hematological toxicity limited systemic treatment to three cycles. Despite further treatment with high-dose cytarabine, he developed progressive neurological manifestations, an unsteady gait, bilateral lower limb weakness and an intention tremor with dysmetria of the right upper arm, and he died 2 years 3 months after the initial diagnosis of neurolymphomatosis.

### Case 3: diffuse large B-cell lymphoma and neurolymphomatosis

**Fig. 4:**
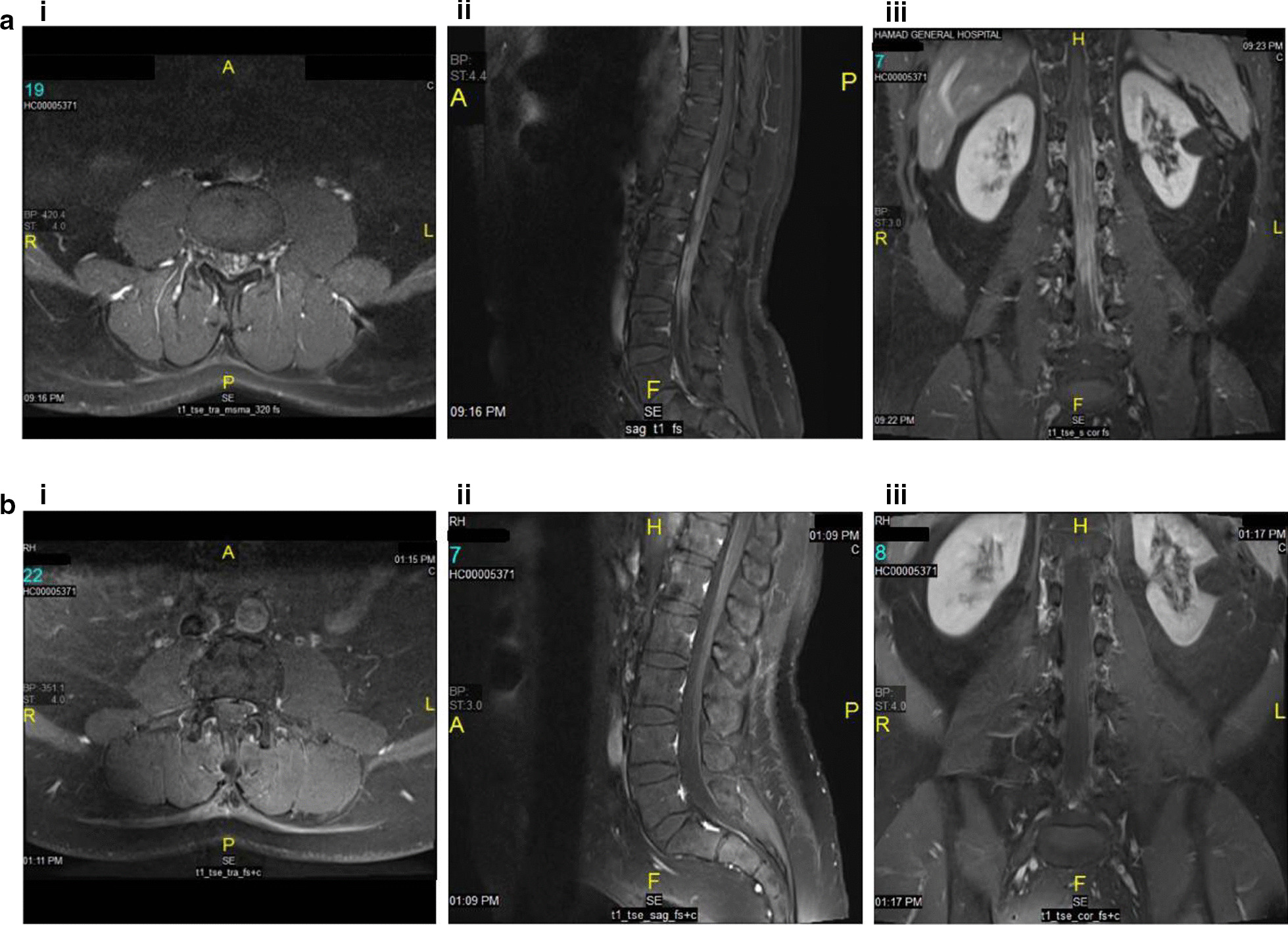
Case 3: radiological findings. **a** Post-contrast MRI studies show thickening and intense enhancement of the cauda equina nerve roots predominantly along the nerve sheath seen extending from the conus medullaris region down into the thecal sac to the lumbosacral plexus likely representing perineural tumor spread. Heterogeneous marrow signal pattern of the imaged vertebrae predominantly involving the lower cervical and upper thoracic vertebral bodies, showing bright signal intensity in T2- and STIR weighted images and heterogeneous mosaic pattern enhancement in the post-contrast series with no evidence of structural collapse or extra-osseous soft tissue component. **b** Post-contrast MRI study 33 months post-autologous stem cell transplant shows almost complete resolution of previously described residual thickening and enhancement of cauda equina nerve roots

A 47-year-old Yemeni man presented with left cervical lymphadenopathy in 2005. Biopsy revealed follicular lymphoma, grade III. Staging workup confirmed an Ann Arbor stage IA disease. He was initially treated with three cycles of R-CHOP followed by involved-field radiotherapy (30 Gy). Eight years later, he relapsed with biopsy-proven DLBCL in the initial anatomical site and stomach fundus. The patient received six R-CHOP cycles with prophylactic intrathecal methotrexate. Following the second complete remission, he received rituximab maintenance for 1 year. Three months following maintenance completion, the patient developed lower limb motor and sensory impairment as well as urinary retention. MRI revealed cauda equina nerve root thickening and enhancement, both intra- and extradural (Fig. 4a, b), suggestive of involvement of the cauda equina by lymphomatous infiltration. CSF examination showed abundant T-cell lymphocytosis (500 cells/µL) and high protein levels. CSF PCR for tuberculosis, Epstein Barr virus and cytomegalovirus was negative. The patient was treated urgently with radiotherapy (10 Gy) to the cauda equina and high-dose intravenous dexamethasone, resulting in significant neurological improvement. A PET-CT scan performed after this treatment showed a diffuse, mild FDG abnormal uptake involving the cauda equina and extending to the conus medullaris. Three cycles of high-dose methotrexate and ifosfamide were administered, followed by high-dose chemotherapy with the BEAM protocol (BCNU, etoposide, cytarabine, melphalan) with autologous stem cell transplantation (ASCT). This treatment resulted in complete remission (Fig. 4c), which is being maintained 40 months post-transplantation.

### Case 4: diffuse large B-cell lymphoma and neurolymphomatosis

**Fig. 5:**
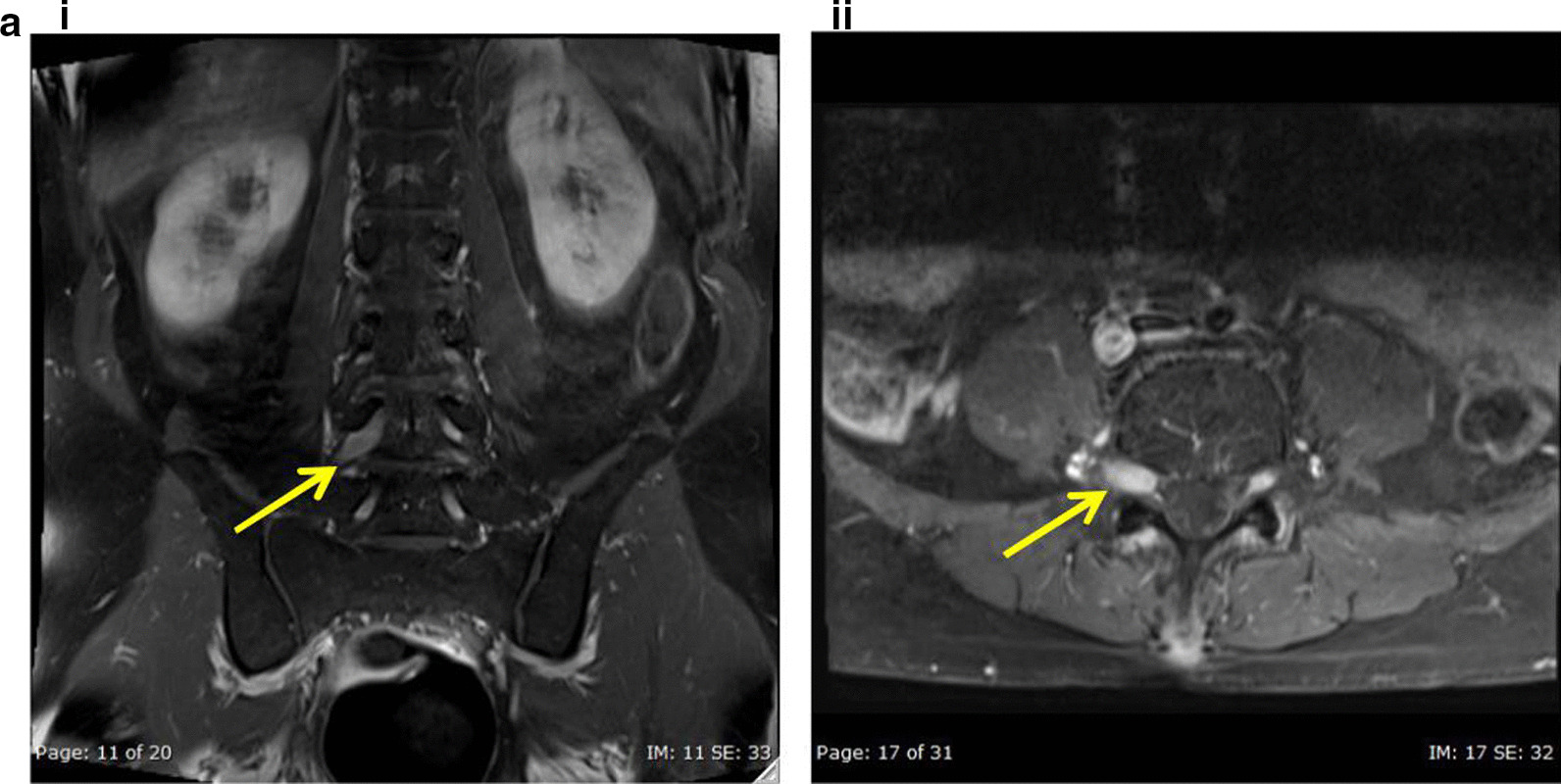
**a** i, ii Coronal and axial post-contrast fat-saturated images show thickening and enhancement of the right L4 nerve root compared to the left one, which is highly suggestive of lymphomatous infiltration (arrow in **a**, **b**)

**Fig. 6:**
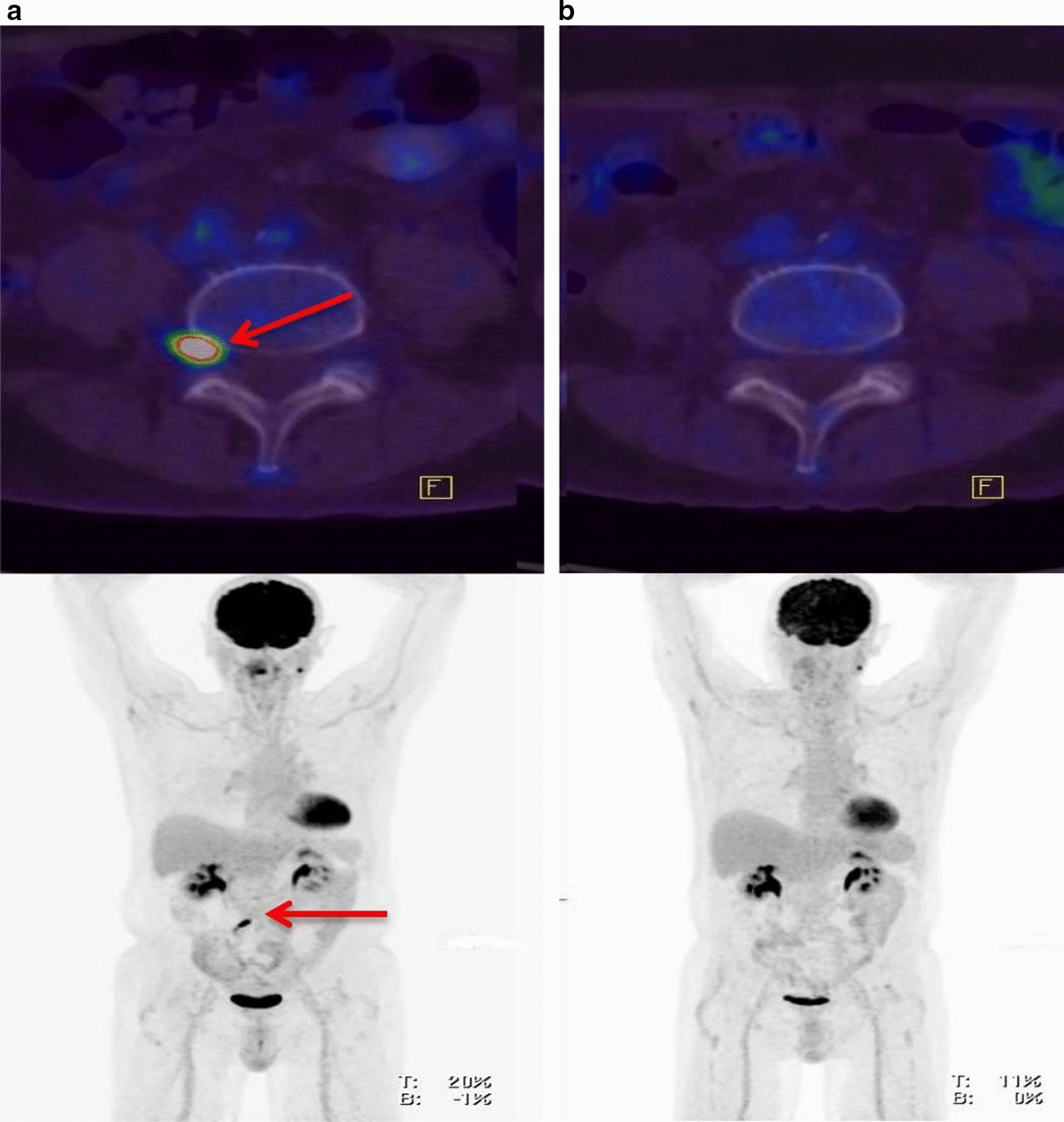
**a** Intense uptake projected to the right L4 spinal nerve root might represent DLBCL relapse (the red arrow is pointing to L4 spinal nerve lesion). **b** Post-treatment: compared to the previous PET/CT complete remission of DLBCL is evident. Left-sided benign tumor of the parotid gland is noted with no interval change

A 66-year-old Moroccan man presented in December 2015 with a large rapidly growing testicular mass and multiple subcutaneous nodules with numbness of the lips and facial asymmetry. Core biopsy from the testis and a subcutaneous lesion showed involvement with DLBCL. Whole-body PET-CT revealed multiple extra-nodal manifestations (testis, skin, paranasal sinus, kidney and adrenal involvement). MRI head was normal, and cerebrospinal fluid cytopathology was negative for malignant cells. The patient achieved complete remission after six cycles of R-CHOP chemotherapy plus intrathecal methotrexate followed by two consolidation courses of high-dose methotrexate and radiotherapy to the paranasal sinuses and both testicles. The patient presented again in May 2017 with right-sided low back pain and severe right lower limb pain. MRI spine showed thickening and enhancement of the right L4 nerve root highly suggestive of infiltration by a neoplastic process (Fig 5). PET-CT revealed intense FDG uptake projected to the right L4 spinal root that likely represented lymphoma relapse, and no other metabolic sign of DLBCL activity was found (Fig. 6a). The patient declined biopsy of the involved nerve root. Salvage chemotherapy with R-DHAP chemotherapy (rituximab, cisplatin, cytarabine and dexamethasone) was initiated. The patient failed to mobilize enough stem cells for autologous stem cell transplantation following his second cycle; however, his PET-CT showed complete remission 4 weeks later. Two further cycles of high-dose methotrexate, thiotepa plus rituximab were given. He maintained complete remission as shown by repeat PET-CT study (Fig. 6b), performed 2 months after completion of his chemotherapy courses. The patient is currently asymptomatic 12 months after his initial diagnosis of NL.

## Discussion and conclusion

For the first time in Arabian Gulf countries and nearby Arab States, to our knowledge, this report describes four patients with neurolymphomatosis. NL has been previously described in adults with a median age of 55.5 years [2], with a male-to-female ratio of 1.5:1. Here, the four patients were all male, with a lower median age of 46.5 (33 to 66) years. The identification of NL in younger patients is predictable in a country where the average age of the population is 31 years old [30]. The disease presentation of these patients shows important peculiarities. The occurrence of NL in the context of Burkitt lymphoma is extremely rare [31]. In this report we describe for the first time, to our knowledge, one patient who developed NL from Burkitt lymphoma presenting as a post-renal transplant lymphoproliferative disorder (PTLD) (case 1). The remaining three patients also displayed interesting clinical presentations, including optic nerve involvement in the context of B-cell acute lymphoblastic leukemia (case 2) and L4 spinal nerve root and cauda equina syndrome in the context of DLBCL (cases 3 and 4).

Nerve biopsy was only performed in one patient (patient 1) in our study. Three patients did not have a biopsy; two refused the procedure (patients 3 and 4), and in one patient the risk of permanent optic nerve injury from the procedure was deemed too high (patient 2). This is in keeping with previous reports where nerve biopsy is only available in about half of cases [1, 2].

MRI and PET/CT can be useful tools to confirm clinical suspicion of NL when a biopsy cannot be obtained. The sensitivity of those two techniques has been compared in previous studies. The International Primary CNS Lymphoma Collaborative Group has shown similarly high sensitivity for MRI and PET/CT (positive findings in 36/47 and 16/19 patients, respectively) [2]. MRI was performed for all four cases described in this report and allowed detection of abnormal findings (enlargement and thickening of the affected nerves and nerve root), strongly supporting the diagnosis of NL in all of them. PET/CT scanning was performed in three patients and was found to be a very useful diagnostic modality, especially in patients 1 and 4 where it complemented the MRI findings and facilitated identification of the affected nerve portion for targeted biopsy (patient 1). In case 3, PET-CT was not used as a diagnostic tool but was performed at a later stage, following radiotherapy and high-dose corticosteroids. SUV uptake was mildly abnormal and heterogeneous, possibly confirming the efficacy of the treatment. However, detection sensitivity of lymphoid tumors by PET/CT can be affected acutely by some treatments, including corticosteroids [32]. In case 3, corticosteroids and radiotherapy may have dramatically reduced tumor metabolism even prior to the regression of the tumor mass. An integrated approach combining MRI and PET/CT may be necessary when a single technique provides limited information.

CSF involvement is not required for a diagnosis of NL. In our report CSF cytopathology findings supported the diagnosis of NL in one case (patient 2) by showing leukemic cells. One further case (patient 3) had increased T lymphocytes in the CSF without any other explanation. All four patients initially relapsed with NL after initial treatment despite complete remission from the original disease.

The initial presenting complaints were varied, including painful peripheral neuropathy (patient 1), cranial nerve palsy (patient 2) and nerve root compression (patient 4) similar to previous descriptions [1], whereas patient 3 presented with cauda equina syndrome. This is uncommon but has been previously reported [13]. In our reported cases NL was caused by aggressive non-Hodgkin lymphoma in three cases (Burkitt lymphoma and DLBCL) and acute B-cell leukemia in one case, this proportion being similar to that described by Grisariu *et al.* [2].

Neurolymphomatosis has not been described before in a patient with post-transplant lymphoproliferative disorder (PTLD). PTLDs are themselves rare complications of transplantation and NL rarer still. PTLDs arise in patients who are chronically immunosuppressed, with an incidence of 1–5% in solid organ transplant recipients and 1% in allogeneic hematopoietic stem cell transplant (HSCT) recipients [33–35]. Most cases of PTLD occur early after the transplantation procedure, often within the first year, but late-onset PTLD can occur [35–38], as seen in our first patient who developed NL 5 years following renal transplant.

Currently, there is no standard treatment for NL; steroids alone provide only short-lived symptom relief [1]. Most patients receive systemic chemotherapy where possible, and this may be combined with intrathecal chemotherapy or radiotherapy, the choice being made according to individual patient characteristics. Intravenous methotrexate in doses exceeding 3.5 g/m^2^ that penetrate the central nervous system can be given, based on experience gained in treating primary central nervous system lymphoma (PCNSL) [39]. Three of our cases were treated with high-dose methotrexate-based chemotherapy, but methotrexate was excluded in patient 1 because of the prior renal transplantation. After salvage therapy with high-dose methotrexate regimen, one patient received high-dose chemotherapy with the BEAM protocol followed by an autologous stem cell transplant (ASCT) (patient 3). This treatment seems to be very effective, as the patient remains in complete remission more than 40 months post-ASCT. Patient 4 achieved a complete remission after two cycles of salvage therapy with DHAP and maintains a CR after high-dose methotrexate and thiotepa. Radiotherapy combined with high-dose steroid was significantly effective in improving neurological symptoms in one patient (patient 3), while combined local radiotherapy with high-dose cyatarabin used in patient number 1, did not change the disease coarse. Rituximab was used in only two patients (patients 1 and 4). It was ineffective in patient 1 but was part of a successful treatment protocol in patient 4.

We conclude that NL is a rare but increasingly recognized entity that can be difficult to diagnose by biopsy but reliably confirmed by a combined imaging approach. However, prior treatment with high-dose dexamethasone with or without local radiotherapy might suppress FDG activity and decrease the sensitivity of PET/CT. The prognosis is generally poor but using high-dose methotrexate as well as high-dose chemotherapy and ASCT may be an effective way to treat NL (Table 1).

**Table 1:** Patient characteristics and outcome

No.	Age	Sex	CT	Steroid	Rituximab	IT	Radiotherapy	ASCT	Outcome
1	33 years	Male	ARA-C	Yes	Yes	No	Yes	No	Expired
2	40 years	Male	ARA-C, MTX, Ifo	No	No	Yes	Yes	No	Expired
3	47 years	Male	MTX, Ifo, and BEAM	Yes	No	No	Yes	Yes	Alive
4	66 years	Male	DHAP, MTX, Thio	Yes	Yes	No	No	No	Alive

*CT* systemic chemotherapy, *IT* intrathecal chemotherapy, *ARA-C* cytarabine, *MTX* methotrexate, *Ifo* ifophosmaide, *BEAM* BCNU, etoposide, cytarabine, melphalan, *ASCT* autologous stem cell transplant, *DHAP* cisplatin, cytarabine, and dexamethasone, *Thio* thiotepa

## Data Availability

All de-identified data are available for external review if needed.

## Abbreviations

FDG: ^18^F-fluorodeoxyglucose
PET/CT: Positron emission tomography/computed tomography
PTLD: Post-transplant lymphoproliferative disorder
B-ALL: B cell-acute lymphoblastic leukemia
NL: Neurolymphomatosis
NHL: Non-Hodgkin lymphoma
MRI: Magnetic resonance imaging
CSF: Cerebrospinal fluid
R: Rituximab
CHOP: Cyclophosphamide_,_ doxorubicin, vincristine and prednisolone
DLBCL: Diffuse large B-cell lymphoma
CR: Complete remission
PCR: Polymerase chain reaction
IHC: Immunohistochemistry
Hyper-CVAD: Hyper-fractionated cyclophosphamide, vincristine, doxorubicin and dexamethasone alternating with high-dose methotrexate and cytarabine
CNS: Central nervous system
BEAM: BCNU, etoposide, cytarabine, melphalan protocol
ASCT: Autologous stem cell transplantation
R-DHAP chemotherapy: Rituximab, cisplatin, cytarabine and dexamethasone
